# Editorial: Cellular contributors and consequences of protein misfolding and aggregation

**DOI:** 10.3389/fmolb.2025.1759495

**Published:** 2026-01-05

**Authors:** Anoop Arunagiri, Emily Sontag, Verena Kohler, Arunkumar Venkatesan

**Affiliations:** 1 Department of Biological Sciences, East Tennessee State University, Johnson City, TN, United States; 2 Department of Biological Sciences, Marquette University, Milwaukee, WI, United States; 3 Department of Molecular Biology, Faculty of Medicine, Umeå University, Umeå, Sweden; 4 Department of Ophthalmology and Visual Sciences, SUNY Upstate Medical University, Syracuse, NY, United States

**Keywords:** endoplasmic reticulum–mitochondria association, liquid–liquid phase separation, neurodegeneration, protein aggregation, proteostasis

Protein function is dictated by its structure, which governs its propensity for self-assembly whether it exists as a folded globular protein or as an intrinsically disordered polypeptide. The material properties of these assemblies range from dynamic liquid-like condensates to solid aggregates. Condensates serve fundamental biological roles, but when regulatory mechanisms fail, it leads to disease. This research explores how proteostasis collapses, driving various proteinopathies, moving beyond fibril formation to uncover complex cellular failures and delineate novel therapeutic strategies.

The core challenge in many neurodegenerative diseases, reviewed by Tsekrekou et al., is the toxicity of soluble oligomers of proteins like TDP-43 and SOD1 in amyotrophic lateral sclerosis (ALS). This underscores the urgent need for mechanism-based inhibitors (e.g., ebselen and QBP1) that stabilize the native protein state or block toxic assembly. This challenge is mirrored in diabetes, as Zavarzadeh et al. reviewed how proinsulin misfolding due to insulin gene mutations (as in mutant INS-gene-induced diabetes of youth, MIDY) or metabolic stress (as in type 2 diabetes) triggers toxic endoplasmic reticulum (ER) stress and the unfolded protein response (UPR). This failure was also identified by Venkatesan and Bernstein as central to glaucoma pathology, where misfolded MYOC and OPTN drive chronic ER stress and mitochondrial dysfunction. However, potential solutions exist. Simha et al. demonstrated that the herb *Sida cordifolia* effectively mitigated Huntington’s disease (HD) pathology in models by specifically reducing mutant huntingtin (mHtt) aggregates and, critically, by suppressing the toxic ER–UPR pathways (PERK, IRE1α, and ATF6).

Beyond genetic and folding defects, the cellular environment plays a crucial role. Da Costa et al. established that mitochondrial inorganic polyphosphate (polyP) is essential for mitochondrial proteostasis, acting as a non-transcriptional regulator that prevents stress-induced protein aggregation (TUFM). Similarly, Suzuki and Itoh highlighted that the synergistic effect of missense mutations and resulting glycosylation defects drives pathology in inherited diseases like CADASIL (NOTCH3) and FH (LDLR), where altered structure near a glycosylation site accelerates misfolding and aggregation. This environmental influence extends to membranes. Schepers et al. reviewed how lipid dysregulation, driven by factors like GBA1 and APOE ε4, is fundamentally linked to the pathology of synucleinopathies (PD and DLB) and dictates the specific alpha-synuclein strains (e.g., Lewy fold vs. MSA filaments) that explain clinical heterogeneity.

Finally, the most unifying concept is the dynamic nature of assembly, which requires new tools for study. Oldam et al. developed a simple thioflavin T in-gel staining method for the quick characterization and quantification of both fibrils and toxic pre-amyloid oligomers (PAOs). Applying this mechanistic understanding, Navalkar et al. synthesized the evidence that the core pathological defect is the failure to maintain the dynamic, liquid material state of protein assemblies, arguing that initially functional liquid–liquid phase separation (LLPS) condensates (like TDP-43 in stress granules or CgB in secretory granules) transition into immobile, microreactor aggregates (like Tau or IAPP) under stress. Ultimately, preserving the liquid-like state of these assemblies emerges as the central therapeutic goal.

